# Bread

**Published:** 1859-07

**Authors:** 


### HALL'S JOURNAL OF HEALTH.

OUR LEGITIMATE SCOPE IS ALMOST BOUNDLESS: FOR WHATEVER BEGETS PLEASURABLE AND HARMLESS FEELINGS, PROMOTES HEALTH; AND WHATEVER INDUCES DISAGREEABLE SENSATIONS, ENGENDERS DISEASE.

*We aim to show how Disease may be avoided, and that it is best, when sickness comes, to take no Medicine without consulting an educated Physician.*

VOL. VI.] JULY, 1859. [No. 7.

### BREAD.

If wheat is pounded or ground very fine, it produces flour, which is composed of two familiar ingredients, starch and glue. In each hundred pounds of fine flour there are ten pounds of this vegetable glue, and seventy pounds of starch. Tapioca, sago, arrowroot, maizena, corn starch, potatoe starch, are all the same thing, only made out of a different material; as sugar is sugar, whether made of cane or sorghum, or beets, or maple tree water; as alcohol, is alcohol whether made of rye, corn grapes, or anything else.

If flour is made up into a lump with water, it is called dough; if then this dough is worked with the hands while water is poured on it, this water is whitened, and if allowed to run into a vessel and settle, a fine powder falls to the bottom, that is the starch; what is left behind, a soft tough substance, is the glue, or gluten. The starch is that which keeps us warm; the gluten nourishes us, gives us flesh. Thus it is, that bread is the staff of life; it strengthens and warms us.

To convert dough into bread, it must have heat applied, which is called baking, or hardening. But for taste and convenience, various plans have been devised to lessen the amount of hardening and solidity, both of which imply heaviness, and in proportion as this heaviness is removed, it is called "light" bread. The lighter it is, the better it is generally considered, although in proportion as it is lighter than that made by simple flour, water and heat, it is at a greater remove from being pure bread. No additions to the simple substances of the whole product of wheat grains mixed with water and baked by fire, can make bread "better," as far as its nutritiousness and wholesomeness are concerned. But here, art interferes to pamper the appetite and make a curse, of what was intended to be a blessing.

Bread is made light, by a mixture of yeast, cream of tartar, saleratus, soda, &c., &c., all of which materials lighten bread in the same way, that is, by producing in the dough, an invisible substance called carbonic acid gas.

If a spark of fire is applied to a grain of powder, it explodes, and there is a product which occupies a much larger space than the grain of powder did. So in a lump of dough, when a particle of cream of tartar touches a particle of soda, and warmth is applied, a volume of carbonic acid gas is the result; and as it is light, it tends to rise, like smoke or steam ; but it is in the midst of a particle of dough ; it makes an effort to rise ; it swells out, and the particle of dough yields a little, but not much, because there is not warmth enough ; just as a partially filled air bladder, it distends, it swells out a little, if a little heat is applied, but it does not burst, because there is not heat enough. This distention results from the philosophical fact, that heat causes bodies to expand, air and gasses especially.

A cup of coffee will dissolve a certain amount of sugar and no more ; all in addition to that goes to the bottom of the cup, and makes the coffee no sweeter.

So in the use of cream of tartar and soda in making bread, a certain quantity of them will mix and give out carbonic acid, and if there be a surplus, that surplus remains in the bread as cream of tartar or soda. One item of skilfulness then, in making good bread, is to put in the exact amount of the articles named ; for in proportion as either is in excess, there is laid the foundation of disease and death.

How many servants or bread bakers in a million will bother themselves with any exactitude as to these points? Hence if the sharp, distinct issue is made, ought these things to be used in bread making? the answer must be a decided and unequivocal no! But how shall we know the proportions of each? That oft abused individual "Science," will answer: If there is too much soda, the bread will be yellow, and the natural acid which is in the gastric juice will be neutralized by it, and it is no longer gastric juice, and as gastric juice is necessary to digestion, digestion is not performed, and the body is harmed. The proper proportion is one ounce of soda and two and a half ounces of cream of tartar; this mixture makes Rochelle salts, half an ounce of which is a cathartic, in the practice of medicine, and enough to make forty ordinary biscuits; so that any person using the soda biscuit of the kitchen, may calculate how many doses of salts he is taking in a year, "for the pleasure of the thing."

Soda and saleratus are made out of ashes. Soda is made of the ashes of vegetation on the sea; saleratus, of vegetation on the land; both contain carbonic acid, which is let loose when cream of tartar is mixed with them, and heat is applied in baking. If there was no glue in the flour, this carbonic acid would nearly all escape, but this glue being tough, yields a little, and a bubble is made, but the bubble does not burst. This bubble takes up room, although it is nothing but thin air, in a sense; hence a loaf of bread is literally a puff; it appears to be more than it is; just like some people. One half of a loaf of bread, as to bulk, is air. As to weight, very near one half is water. In a hundred pounds of common light bread there are forty pounds of water; even if bread is several days old, it has almost as much water in it as when first baked, so that "stale bread" is no drier than fresh bread, for if put in a closed tin vessel, and then placed in a heated oven, it becomes as soft as when first baked. In what manner or condition the water is concealed in the bread, chemistry has not yet found out.

Yeast answers the same purpose as cream of tartar and soda, for these last have the acid and potash and carbonic acid. Yeast alone has all these. As soon as the dough in which these articles have been mixed is placed in a heat of from seventy to ninety degrees, it begins to "rise," that is, it begins to be puffed up by the globules of carbonic acid which are let loose; we call it fermentation; it is decomposition; it is the first step towards destruction or putrefaction, or rotting; all of which would take place in time, if this moderate heat were not put a stop to by the greater heat of the oven which "sets" the bread; that is, arrests the throwing off of carbonic acid; the hard crust on the outside of the loaf keeping it within the loaf in spite of the greater heat.

Whenever bread is sour, it is because the fermentation had continued too long, or the heat was too great, great enough to burst the little vesicles which contained the carbonic acid gas; and on their bursting, the bread particles closed in upon the space left, and instead of "lightness" there is solidity, and we call it "heavy," "sodden."

The "leaven" of scripture is the yeast of our own day. "A little leaven leaveneth the whole lump." A very little yeast makes a large lump of dough to rise, because it diffuses itself through the whole mass of dough with wonderful rapidity; as wonderful as Jonah's gourd, or the mushroom of a night. Had we time to brush up our memories as to Hebrew "roots," we might find out, or fancy, that a gourd and a toadstool were of the same derivation; at all events, they grow in a night, and perish with a breath. In fact, science goes on to say that toadstools, mushrooms, the green mould on sour paste, spoiled preserves and yeast, are identically of the same nature; a species of vegetation that multiples upon itself; feeds upon itself, and also upon what it attaches itself to; and that each atom of mould, as soon as it is detached, is in the nature of a seed, and begins instantly to fructify wherever it lights, if there be only adequate warmth and moisture. The pollen of a flower or tree has lighted on ships far out at sea, and yet these are six times larger than an atom or seed of mould or mildew, so that when warmth and moisture and stillness of atmosphere all favor, it is no wonder that mildew begins to pervade every cranny of space; and if not arrested by greater heat, or cold, or dryness, universal decay and death would be the sad ending. So, familiar yeast, like familiar fire and water, is an enemy or friend according as kept under control.

Perhaps after all, the reason why the eating of ship biscuit is an almost indispensible aid in the cure of certain forms of aggravated dyspepsia, is the patient lives on pure bread, and does not fill his stomach with acids and alkalies; with cream of tartar and Rochelle salts; with toadstools, mushrooms, white mildew, green moulds and the like things, the very thought of which is nauseating.

If men were not disposed to abuse their liberties; and if it had not been found that in the temperance reformation an occasional dram was fatal to the whole thing; and if our cooks had intelligence enough to use the various kinds of "risings" judiciously; and if we would eat our nice warm biscuits slowly, and in moderation, say two or three at a meal, we would say that an occasional indulgence of "hot cakes," the "biscuits" of old times, at a winter's breakfast, or on emergency, once or twice a week, might be allowable, and that no appreciable injury would result from such indulgence in the course of a lifetime, where the person thus indulging was in pretty good health. To others, the common ship biscuit is the best for a stand-by. There are few forms of bread really better, if just before being used, boiling water is poured on them and they are covered over a few minutes in such a way as to keep the steam in.

Some bakers use an ounce of alum in a hundred pounds of flour; in such proportion it lightens and whitens the bread, and which the baker very well knows, enables a loaf to retain more water, so he gets more money and his customers less bread.

Lime-water is sometimes used to improve bad flour—about four gallons to a hundred pounds of flour—that is about four grains of lime in a pound of flour, or about three grains in a loaf of bread claiming to weigh a pound.

Homœpathy claims that the more extensively an atom of medicine is diluted or comminuted in water, the more powerful it is. If the same holds good as to flour, we may say that the amount of either alum or lime in bread, as above, is enough to have a tremendous effect on everybody, and the reason that everybody is not dead long before now is simply that everybody is all the time eating "incompatible" things, which nullify infinitesimals, and the result is that people are just about in the same condition as if they had not taken lime, alum, soda, saleratus, cream of tartar, mushrooms, yeast, mildew, and toadstools, and therefore none of these things have hurt anybody, as far as bread eating is concerned. Homœpathy therefore contains a grand truth or a most tremendous absurdity; meanwhile we will luxuriate in the middle track, and believe that Homœpathy allows some people to get well, and that saleratus has not killed everybody.

One item as to bread making should not be forgotten. The

French make the best bread in the world; this has been attributed to their regulating the heat of their ovens by a thermometer; too little heat makes the bread sodden and sour; too much burns the outside while the inner part is not cooked; the intensity and duration must be found in observation and experiment.

